# Neurocognitive development in isolated Robin sequence treated with the Tuebingen palatal plate

**DOI:** 10.1007/s00784-022-04448-3

**Published:** 2022-03-19

**Authors:** Andreas Naros, Irene Steiner-Wilke, Nadja Kaiser, Margit Bacher, Bernd Koos, Gunnar Blumenstock, Cornelia Wiechers, Christian F. Poets, Siegmar Reinert, Michael Krimmel

**Affiliations:** 1 Department of Oral and Maxillofacial Surgery, Tuebingen University Hospital, Osianderstrasse 2-8, 72076 Tuebingen, Germany; 2 Centre for Cleft Palate & Craniofacial Malformations, Tuebingen University Hospital, Tuebingen, Germany; 3 Department of Paediatric Neurology and Developmental Medicine, University Children’s Hospital Tübingen, Tuebingen, Germany; 4 BIP-Orthodontic Practice, Tuebingen, Germany; 5 Department of Orthodontics, Tuebingen University Hospital, Tuebingen, Germany; 6 Department of Clinical Epidemiology and Applied Biometry, University of Tuebingen, Tuebingen, Germany; 7 Department of Neonatology, Tuebingen University Hospital, Tuebingen, Germany

**Keywords:** Robin sequence, Cleft palate, Tuebingen palatal plate, Pre-epiglottic baton plate, Neurocognitive development, Wechsler Pre-school and Primary Scale of Intelligence (WPPSI-III), Kaufman Assessment Battery for Children (K-ABC)

## Abstract

**Objectives:**

We aimed to determine the neurocognitive development of cleft palate patients with and without Robin sequence (RS).

**Materials and methods:**

Children with isolated RS with cleft palate and children with cleft palate only (CPO) were contacted at the age of 5–6 years. All RS children had undergone initial polygraphic sleep study (PG) with a mixed-obstructive apnea index (MOAI) of ≥ 3/h and were consequently treated with the Tuebingen palatal plate. A standardized clinical examination as well as a neuropediatric and neuropsychological examination included the Wechsler Pre-school and Primary Scale of Intelligence (WPPSI-III), Kaufman Assessment Battery for Children (K-ABC), and an assessment of developmental milestones.

**Results:**

In total, 44 children (22RS, 22CPO) were included. RS children were younger at study (70.5 ± 7.3 and 75.2 ± 7.5 months; *P* = .035). Both groups achieved the evaluated milestones within the normed time frame. WPPSI-III and K-ABC results showed no group differences. Mean values for Verbal IQ (101.8 ± 11.1 vs. 97.1 ± 15.7), Performance IQ (102.9 ± 12.1 vs. 99.6 ± 14.5), Processing Speed Quotient (98.9 ± 15.6 vs. 94.5 ± 15.7), Full-Scale IQ (103.2 ± 12.1 vs. 98.4 ± 15.3), and Sequential Processing Scale (102.1 ± 13.1 vs. 94.2 ± 17.3) were within the reference range (IQ 85–115) for RS and CPO children, respectively, indicating average performance of both groups.

**Conclusion:**

No neurocognitive, physical, or mental impairments were detected suggesting that RS children having upper airway obstruction (UAO) treated early and effectively may use their potential for an age-appropriate neurocognitive development.

**Clinical relevance:**

Tuebingen palatal plate treatment successfully releases UAO. Thus, isolated RS does not necessarily result in developmental delay or an impaired neurocognitive outcome.

**Trial registration:**

Deutsches Register Klinischer Studien, DRKS00006831, https://www.drks.de/drks_web/

## Introduction

First described by the French stomatologist Pierre Robin in 1923, the eponymous sequence (RS) is defined as a triad of micrognathia, glossoptosis, and upper airway obstruction (UAO) [1]. A rather wide, sometimes u-shaped cleft palate appears in up to 85% of patients [2, 3]. However, clefting is only considered facultative for the diagnosis. Reported incidence rates of RS vary between 1:8500 and 1:14,000 [2, 4, 5]; up to 50% are associated with other syndromes [2, 6]. Due to the associated UAO, RS is a potentially life-threatening condition. Developmental delay and failure to thrive may result from recurrent hypoxemia and feeding problems [3]. The main focus therefore should be on the early treatment of UAO and feeding problems. Various surgical and non-surgical treatments have been developed. However, there is a lack of consensus regarding the best treatment for UAO. Moreover, high-level evidence on treatment outcome is scarce [3, 7]. We established an interdisciplinary non-invasive treatment protocol including the early application of the Tuebingen palatal plate (TPP), also known as pre-epiglottic baton plate (PEBP), in combination with additional intensive feeding training and functional orofacial regulation therapy (Castillo Morales). The TPP consists of a plate with velar extension that pushes the tongue anteriorly, thereby widening the hypopharynx and releasing the UAO [8]. The effectiveness of this method has been extensively studied in prospective mono- and multicentre studies, in isolated and syndromic RS patients [8–11] as well as in children with syndromic craniosynostosis and sleep-disordered breathing (SDB) [12].

Several authors reported neurocognitive deficits in RS patients [13, 14]. However, whether and to what extent RS has a direct impact on patients’ cognition is still unclear [3, 15, 16].

We aimed to investigate the neurocognitive, physical, and mental development of children with isolated RS with cleft palate at 5–6 years of age. We hypothesized that RS patients treated with the TPP have an age-appropriate development without neurocognitive deficits, since TPP treatment successfully releases UAO.

## Material and methods

### Study design

In this prospective observational study (DRKS00006831), two groups of cleft patients were compared at age 5–6 years. Group 1 (RS) consisted of patients with isolated RS with cleft palate, all diagnosed by pre-therapeutic polygraphic sleep study (PG) with a mixed-obstructive apnea index (MOAI) ≥ 3/h. Group 2 contained patients with cleft palate only (CPO). Inclusion criteria were as follows: (i) age at survey 5–6 years; (ii) cleft palate; (iii) cleft surgery within the first 18 postnatal months; and, in RS group only, (iv) initial MOAI > 3/h and TPP treatment. All patients with comorbidities, additional syndrome(s), or sequences were excluded.

This study was approved by the institutional ethics committee (586/2014B01), and written informed consent given by parents.

### Interventions

All study-related interventions were carried out on one day, under standardized conditions (time, environment) and by the same investigators. First, the aftercare and clinical examination took place. Hereby, clinical characteristics of patients as well as the current respiratory situation and clinical signs of persisting UAO (i.e., snoring, thoracic retractions, sweating during sleep, daytime sleepiness) were documented.

Furthermore, a specialist neuropediatric examination was performed to exclude neurological disorders, i.e., spastic, dyskinetic, or atactic movement disorders defined according to the Surveillance of Cerebral Palsy in Europe (SCPE) criteria, as well as children with unexpected concomitant diseases or syndromes. Also, the questionnaire on developmental milestones (i.e., gross motor skills and linguistic milestones) was completed together with the parents. These milestones have been defined and validated as reliable screening tests to assess early childhood development [17, 18]. To achieve the most accurate information possible, questionnaires were sent to parents by mail at least 4 weeks before the day of study. Results are provided as mean ± SD and also as absolute data in three rated categories (i) timely development, (ii) developmental delay (by < 12 months), and (iii) developmental disorder (> 12 months delay) compared to German reference values [17].

Neuropsychological examinations were performed by an experienced developmental psychologist. Neuropsychological testing included the Wechsler Pre-school and Primary Scale of Intelligence—Third Edition (WPPSI-III) [19] and the Kaufman Assessment Battery for Children—sequential processing scale (K-ABC-SPS) [20].

The WPPSI-III provides an overall IQ as a measure of the cognitive developmental status of a pre-school child. The WPPSI-III consists of 14 subtests that can be divided into three groups: Core Subtests (7), Supplemental Subtests (5), and Optional Subtests (2). In this study, the Core Subtests were performed, i.e., the verbal part (“Information,” “Vocabulary,” and “Word Reasoning”), the performance part (“Block Design,” “Matrix Reasoning,” and “Picture Concepts”), and the “Coding.” To obtain a Processing Speed Quotient, the supplemental sub-test “Symbol Search” was also performed. Tests were conducted in a highly standardized manner, ensuring high reliability and comparability. Raw values were converted into standard values using tables. These scale scores were then transformed into IQ values for the Verbal IQ (VIQ), the Performance IQ (PIQ), the Processing Speed Quotient (PSQ), and the Full-Scale IQ (FSIQ). The VIQ is a measure of acquired knowledge, verbal reasoning, and comprehension of verbal information; the PIQ measures a child’s nonverbal reasoning, spatial processing skills, attentiveness to detail, and visual-motor coordination skills. The PSQ provides a measure of the ability to quickly and correctly scan, sequence, and discriminate simple visual information. The FSIQ is considered the most representative measure of general intellectual abilities [19]. The split-half reliability varies on the sub-test level between *r* = 0.77 and *r* = 0.88 and on the index level between *r* = 0.87 and *r* = 0.92. For the overall test, it amounts to *r* = 0.95. For the assessment of short-term memory, the K-ABC-SPS, an intelligence test for children between 4 and 12 years of age, was additionally determined [20], consisting of the following subtests: (i) word order, (ii) number recall, (iii) hand movements. Tests were carried out in a highly standardized manner. Raw values were also converted into standard values using tables. From the three subtests, an overall value for the SPS was calculated. The average reliability for the SPS scale between 5 and 12.5 years of age is *r* = 0.88 according to the interpretation manual.

### Statistical analysis

Statistical analysis was performed using JMP® Pro 14.2 (SAS Institute, Cary, NC, USA). Figures were created using GraphPad Prism 7 (GraphPad Software, San Diego, CA, USA). Categorical data are reported as numbers and percentages; continuous data are summarized with the mean and standard deviation if not otherwise indicated. The normality assumption was checked both graphically and by using the measures of skewness and kurtosis. Data are visualized with stacked bar charts and with Tukey’s box-and-whisker plots, respectively. The two-tailed Student *t* test was used to compare normally distributed continuous variables in RS and in CPO children, and the one sample *t* test was performed to test for a deviation from specified norm test values. Considering the limited sample size situation, the two-sided Fisher’s exact test was carried out to compare categorical outcomes between the two study groups. *P* values ≤ 0.05 were assumed to reflect statistical significance. A sample size of 22 in each group will have 80% power to detect an effect size of 0.875 using a two-group *t* test with a 5% two-sided significance level. Developmental milestones were categorized as timely if within the 90th centile norm values, as delayed in case of a ≤ 12 months delay, and as a disorder if delayed by more than 12 months. Neuropsychological test results are provided as Tukey box-and-whisker plots (whisker 1.5*IQR) as well as absolute data in three rated categories, (i) average development (IQ 85–115), (ii) below average (IQ < 85), and (iii) above average (IQ > 115).

## Results

**Fig. 1:**
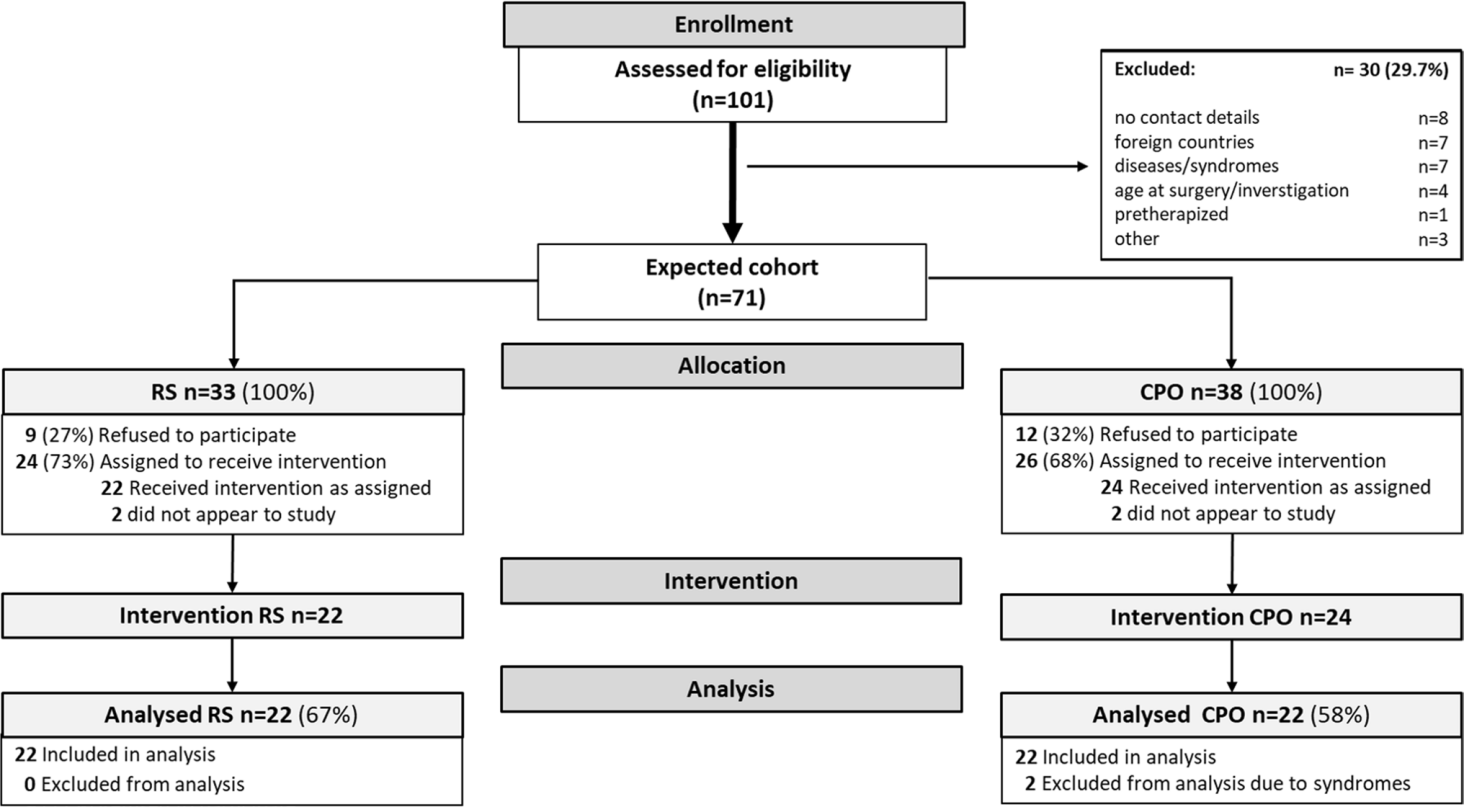
CONSORT flowchart of patient recruitment. RS = Robin sequence, CPO = cleft palate only

In the initial enrollment, 101 children, born between 04/2008 and 04/2013, were assessed for eligibility; 65 had to be excluded for various reasons (Fig. 1). Ultimately, 44 children (20 male, 24 female) were included in this study. All included children in both groups suffered from cleft palate only. Their gender and their surgeons were equally distributed in both groups. RS children were significantly older at cleft palate surgery (11.8 ± 2.7 vs. 7.1 ± 2.2 months; *P* < 0.001), but significantly younger at study (70.5 ± 7.3 vs. 75.2 ± 7.5 months; *P* = 0.035). Included children were all treated in our center and operated by one of two highly experienced surgeons. Mean MOAI in the initial polygraphic sleep study (PG) was 21.2 ± 18.2/h (mean ± SD); thus, all RS patients received TPP treatment. Within the first hospital stay lasting 13.3 ± 8.6 days, the MOAI was significantly reduced (*P* < 0.001) to near-normal values (1.65 ± 1.9/h).

Concerning their current respiratory situation, more snoring was reported in RS children compared to CPO group (RS *n* = 9 (40.9%), CPO *n* = 4 (18.2%); *P* = 0.19). No differences were seen concerning night sweating (RS *n* = 3 (13.6%), CPO *n* = 2 (9.1%); *P* < 0.99) or daytime sleepiness (RS *n* = 2 (9.1%), CPO *n* = 1 (4.5%); *P* > 0.99). No thoracic retractions were reported in any child.

The evaluation of the gross motor skills and linguistic milestones showed that both groups achieved the evaluated milestones within the 90th centile of the reference range (Table 1). However, rated categories revealed more children in the CPO group having a delayed speech development (*n* = 6, 27.3%) and/or a developmental speech disorder (*n* = 3, 13.6%) compared to the RS group (delay, *n* = 6 (27.3%); disorder, *n* = 1 (4.5%); *P* = 0.73). Concerning gross motor skills similar results were seen in both groups (timely, *n* = 19 (86.4%); delay, *n* = 3 (13.6%), disorder *n* = 0 (0.0%); *P* > 0.99).

**Table 1:** Developmental milestones

Milestones	Norm values	RS	CPO
Motor			
Fist handle; reaches for objects	5–6	4.0 (± 1.4)	4.3 (± 1.6)
Moving in prone position (e.g., turning, crawling)	9	7.0 (± 2.1)	7.4 (± 2.2)
Free sitting	10	8.9 (± 2.3)	8.2 (± 1.5)
Free safe walking	18	15.4 (± 2.7)	14.0 (± 2.2)
Short one-legged stand	36	33.4 (± 9.1)	34.3 (± 7.9)
Walking stairs freehand and alternating	48	40.7 (± 12.5)	40.2 (± 7.2)
Stands 5 s on one leg	60	51.5 (± 10.5)	53.7 (± 10.0)
Speech			
Syllable chains (wawawa)	9	8.2 (± 3.8)	9.1 (± 2.9)
“Mama” and “papa”| analogous + 1 word	18	13.9 (± 4.5)	15.0 (± 5.5)
2-word sentences; shows several body parts	24	22.3 (± 8.5)	23.8 (± 7.2)
Recounts longer story; good grammar	48	46.9 (± 12.8)	45.9 (± 12.3)

Values are provided as mean (± SD) (months). Norm values refer to the 90th centile in Germany. No statistical significance (*P* > 0.05)

**Fig. 2:**
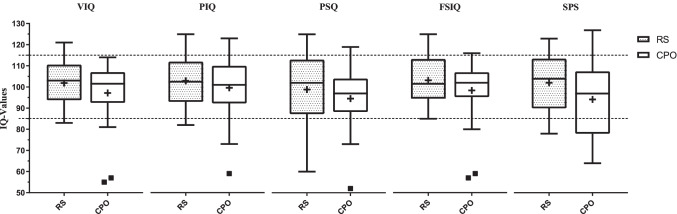
Neuropsychological results from WPPSI-III and K-ABC are provided as Tukey box-and-whisker plots (whisker = 1.5*IQR, mean [** +**] and median). Verbal IQ (VIQ), Performance IQ (PIQ), Processing Speed Quotient (PSQ), Full-Scale IQ (FSIQ), Sequential Processing Scale (SPS). Reference range (IQ 85–115) is displayed as dotted horizontal lines. No statistical significance (*P* > 0.05)

**Fig. 3:**
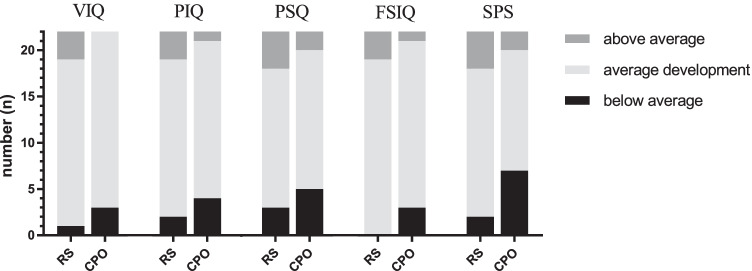
Rated neuropsychological outcome. Evaluation of the neuropsychological outcome in rated categories. Average development (IQ 85–115), below-average performance (IQ < 85), above-average performance (IQ > 115). No statistical significance (*P* > 0.05)

Neuropsychological results from WPPSI-III and K-ABC-SPS showed no group differences (Table 2). Furthermore, mean values for VIQ, PIQ, PSQ, FSIQ, and SPS were within the reference range (IQ 85–115) indicating an average performance of the children investigated (Fig. 2). However, regarding the rated categories for neuropsychological outcome, below-average performance (IQ < 85) was more likely seen in the CPO group whereas above-average performance (IQ > 115) was more likely seen in the RS group (Fig. 3), but none of these differences was statistically significant (*P* > 0.05).

**Table 2:** WPPSI-III and K-ABC-SPS results

	RS	CPO	*P*-value
VIQ	101.8 ± 11.1	97.1 ± 15.7	*P* = 0.26
PIQ	102.9 ± 12.1	99.6 ± 14.5	*P* = 0.42
PSQ	98.9 ± 15.6	94.5 ± 15.7	*P* = 0.37
FSIQ	103.2 ± 12.1	98.4 ± 15.3	*P* = 0.26
SPS	102.1 ± 13.1	94.2 ± 17.3	*P* = 0.09

WPPSI-III and K-ABC-SPS results are provided as mean ± SD. *VIQ* Verbal IQ, *PIQ* Performance IQ, *PSQ* Processing Speed Quotient, *FSIQ* Full-Scale IQ, and *SPS* Sequential Processing Scale are displayed. No statistical significance (*P* > 0.05)

## Discussion

The UAO associated with RS makes it a potentially life-threatening and debilitating condition. Therefore, early treatment is of utmost importance. There is still a lack of consensus, however, regarding the best treatment options for UAO, partly due to a lack of high-level evidence on treatment results [3, 7]. Numerous options are described in the literature, ranging from prone positioning, nasopharyngeal tube, and continuous positive airway pressure (CPAP) to more invasive procedures such as tongue-lip adhesion, mandibular distraction osteogenesis, or tracheotomy [3, 7, 16, 21]. However, TPP yet represents the only therapeutic option for UAO whose efficacy has been demonstrated in a prospective randomized clinical trial [8].

In the current literature, it remains controversial whether and to what extent isolated RS has a direct impact on a patient’s cognition, but a systematic review clearly showed that intermittent hypoxia has negative effects on cognition in children [22]. Moreover, Abadie et al. [14] suggested a prenatal and neonatal brainstem dysfunction as an underlying neuroembryological impairment in RS. In the early 1990s, Caouette-Laberge et al. [13] reported a large series of 125 RS patients treated between 1964 and 1991 with a high proportion of psychomotor impairment and mental retardation (23.1%). This was true also for moderate cases (20%) and was associated with a high mortality rate (16.6%). The authors suggested that improved surveillance and early intervention would improve outcome, at least in children with isolated RS [13].

Regarding the mental development of cleft palate children, a study on 180 children, 14 of these with RS, evaluated the mental development index (MDI) using the Bayley Scales of Infant Development at age 4–36 months [23]. RS children had a mean MDI in the lower range and significantly lower than those with other cleft types. However, neither the management of UAO nor the phenotypes of the RS children was described [23]. Moreover, Persson et al. [24] reported that individuals with RS experienced difficulties in their educational achievements in compulsory school in Sweden. At age 16 years, individuals with RS (*n* = 68) more often did not receive their leaving certificate (9.68%) compared to age-matched controls (2.74%, *n* = 1,249,404) [24].

In a longitudinal prospective study on psychomotor and cognitive, speech, and eating behavior outcomes of 39 severe RS cases evaluated at 15 months, and 3 and 6 years, cognitive scores were within the reference range and increased over time, suggesting good long-term development and prognosis for children with isolated RS. Long-term outcome was independent of the chosen treatment approach (53.8% prone positioning and nasopharyngeal intubation, 46.2% tracheotomy) [25]. In contrast to Persson et al. [24], no difficulties in educational achievements were reported at age 11–12 years. However, 3 children (12.5%) had fallen behind by 1 year of age and another 3 (12.5%) were in a special education program [25]. At 6 years of age, the reported mean MPC score was 109.1 ± 23.9 and the K-ABC-SPS was 110.5, which is slightly higher than our cohort’s results (SPS = 102.1). According to the authors, these results justify a more invasive treatment protocol, assuming that this will protect these children’s cognitive potential [25]. However, we have shown in previous studies that TPP treatment, as a less invasive treatment option, sufficiently releases UAO, preventing the occurrence of intermittent hypoxia in both isolated and syndromic RS [8–11].

In another cohort from our center, we investigated the cognitive and psychosocial development of children with isolated RS (*n* = 34, 4–11 years) also treated with the TPP. Their K-ABC results were within the reference range, but RS children scored slightly lower than healthy controls. No major cognitive impairments were seen in that cohort [15].

In the present study, we aimed to focus on the relevant pre-school age of 5–6 years, i.e., included a narrower age range. We consider this to be the crucial age for functional and neurocognitive development in cleft patients with or without RS, as children enter school and start competing with their peers. Here, it is reassuring to note that with only few exemptions, children reached average (RS *n* = 19; CPO *n* = 18) or even above-average (RS *n* = 3; CPO *n* = 1) FSIQ values. Since the FSIQ is considered the most representative measure of general intellectual abilities, we conclude that there is no cognitive impairment of children with isolated RS at the relevant age of 5–6 years after TPP treatment.

In our cohort of patients, children in CPO group were significantly younger at surgery (11.8 ± 2.7 vs. 7.1 ± 2.2 months; *P* < 0.001). There is growing concern about the effects of early general anesthesia on neurodevelopment in children. However, latest research questions this hypothesis, at least for single anesthesia, as present in our study [26]. Furthermore, our present data underline that both groups have no neurocognitive, physical, or mental impairments regardless of the time of operation.

Nonetheless, it cannot be completely excluded that some results have been subject to bias. Since a normal outcome can be expected in children with CPO, possibly a selection bias occurred. Parents of children with difficulties at pre-/school age may have been more willing to participate in our study than those whose children did not show any signs of impairment. Moreover, due to the severity of the disease, parents of RS children are sensitized to the issue from the very beginning. Thus, in our experience, RS children receive an increased and targeted level of support. Furthermore, the socio-economic background of the participating families was not taken into account.

## Conclusion

In conclusion, isolated RS does not necessarily result in developmental delay or an impaired neurocognitive outcome. By an early and successful release of UAO, affected children may use their potential to have an age-appropriate neurocognitive development.
